# Changes in the Lipid Metabolism of the *Longissimus thoracis* Muscle in Bulls when using Different Feeding Strategies during the Growing and Finishing Phases

**DOI:** 10.3390/metabo13101042

**Published:** 2023-09-28

**Authors:** Juliana Akamine Torrecilhas, Guilherme Luis Pereira, Elias San Vito, Giovani Fiorentini, Germán Darío Ramirez-Zamudio, Larissa Simielli Fonseca, Rodrigo de Nazaré Santos Torres, Tiago Adriano Simioni, Juliana Messana Duarte, Otavio Rodrigues Machado Neto, Rogério Abdallah Curi, Luis Artur Loyola Chardulo, Welder Angelo Baldassini, Telma Teresinha Berchielli

**Affiliations:** 1 School of Veterinary e Animal Science (FMVZ), São Paulo State University (Unesp), Jaboticabal 14884-900, SP, Brazil; juliana.akamine@unesp.br (J.A.T.); rodrigo.torres@unesp.br (R.d.N.S.T.); otavio.machado@unesp.br (O.R.M.N.); rogerio.curi@unesp.br (R.A.C.); luis.artur@unesp.br (L.A.L.C.); w.baldassini@unesp.br (W.A.B.); 2 Confina Beef Cattle Consulting, Sinop 78555-603, MT, Brazil; sanvitoelias@gmail.com; 3 Department of Animal Science, Federal University of Pelotas (UFPEL), Pelotas 96160-000, RS, Brazil; giovani.fiorentini@ufpel.edu.br; 4 College of Animal Science and Food Engineering (FZEA), University of São Paulo (USP), Pirassununga 13635-900, SP, Brazil; germanramvz@usp.br; 5 School of Agriculture and Veterinary Sciences (FCAV), São Paulo State University (Unesp), Jaboticabal 14884-900, SP, Brazil; larissa.simielli@unesp.br (L.S.F.); simioni@zootecnista.com.br (T.A.S.); juliana.d.messana@unesp.br (J.M.D.); telma.berchielli@unesp.br (T.T.B.)

**Keywords:** beef cattle, lipogenic genes, marbling, meat quality

## Abstract

The objective was to evaluate the supplementation strategy’s effect on beef cattle during the growing phase and two systems during the finishing phase. One hundred and twenty young bulls were randomly divided in a 2 × 2 factorial design to receive either mineral (ad libitum) or protein + energy (3 g/kg body weight (BW)/day) during the growing phase and pasture plus concentrate supplementation (20 g/kg BW/day) or feedlot (25:75% corn silage:concentrate) during the finishing phase. Feedlot-fed bulls had meat (*Longissimus thoracis*—LT) with a higher content of lipids and saturated and monounsaturated fatty acids and a greater upregulation of *stearoyl-CoA desaturase* and *sterol regulatory element-binding protein-1c* than animals that fed on pasture (*p* < 0.05). On the other hand, pasture-fed bulls had meat with a higher content of α-linoleic acid, linolenic acid, and n6 and a greater n6:n3 ratio compared to the feedlot-fed group (*p* < 0.05). In addition, meat from pasture-fed bulls during the finishing phase had 17.6% more isocitrate dehydrogenase enzyme concentration than the feedlot group (*p* = 0.02). Mineral-fed and pasture-finished bulls showed down-regulation of *peroxisome proliferator-activated receptor gamma* (*p* < 0.05), while the bulls fed protein + energy and finished in the feedlot had higher *carnitine palmitoyltransferase 2* expression (*p* ≤ 0.013). In conclusion, mineral or protein + energy supplementation in the growing does not affect the fatty acid composition of intramuscular fat of LT muscle. In the finishing phase, feeding bulls in the feedlot upregulates the lipogenic genes and consequently improves the intramuscular fat content in the meat.

## 1. Introduction

In the growing phase of beef cattle, supplementation strategies are used to increase the efficiency of the grazing system and animal performance since tropical forages barely meet the nutritional requirements of animals. Furthermore, the growing phase is a critical point in reducing the slaughter age in response to better biological efficiency in tissue deposition in young animals [1]. In this sense, most studies have evaluated the effect of supplementation during the growing phase on the finishing performance of cattle [2,3,4]. However, little is known about how skeletal muscle metabolism is affected by these nutritional strategies and their response to meat quality, especially intramuscular fat deposition, a criterion of great importance in several countries.

Fat deposition is a response to the activation of metabolic pathways that control uptake, synthesis, and lipolysis, which occur according to the need for lipid release or storage and are regulated by the interaction of dietary nutrients and the expression level of genes involved in lipid metabolism [5]. Animal supplementation with concentrated diets may increase the amount of insulin in the blood, which stimulates glucose uptake by tissues, consequently increasing the amount of intramuscular lipids [6]. Such conditions could increase the expression of genes such as *SCD-1*, which is associated with the conversion of saturated fatty acids into monounsaturated. Greater expression of *SCD-1* is dependent on metabolic signals such as glucose and insulin in the blood, as reported [7].

The use of a high-concentrate diet in cattle feed is important during the finishing phase and can reduce the feedlot period and improve carcass fat deposition. Although the cattle finished in the pasture system supplemented with higher concentrate (1.5 to 2% of body weight [BW]) had higher nutrient requirements than those in the feedlot system [8], this system can be used alternatively to produce carcasses with minimum cover fat [9] as the operational costs of the system may decrease. However, meat from cattle supplemented with grain feed is known to have a greater amount of saturated fatty acids (SFAs) and a less favorable n6/n3 ratio than those from cattle fed exclusively with grass [10]. Although previous works have investigated the beef quality in different systems [11], there are no studies that evaluate the effect of supplementing with a high-concentrate diet (2% BW) on the intramuscular fat and fatty acid profile of meat from cattle finished in pasture systems (2% BW).

It was hypothesized that the supplementation strategy in the growing, followed by feedlot finishing, would influence skeletal muscle metabolism by regulating lipogenic genes, which may impact intramuscular fat deposition. In contrast, finishing bulls on pasture with high concentrate supplementation will have higher unsaturated fatty acid content and a more favorable n6/n3 ratio than animals finished in a feedlot system. In this context, the objective of this study was to evaluate the effect of the supplementation strategy during the growing and finishing phases (pasture supplemented with concentrate or conventional feedlot) on the fatty acid profile, lipogenic enzyme activity, and relative abundance of mRNA associated with lipid metabolism in the *Longissimus thoracis* (LT) muscle of beef cattle.

## 2. Materials and Methods

This study was approved by the Ethics and Animal Welfare Committee of São Paulo State University (protocol 5628/15). The study was carried out at the beef cattle facility of São Paulo State University, Jaboticabal, São Paulo, Brazil (48°1858′ W, 21°15 22′ S).

### 2.1. Animals

The experimental period comprised the growing phase (first experimental period) and the finishing phase (second experimental period). The study was conducted between December and September (285 d). One hundred and twenty bulls from three genetic groups: 40 Nellore (10 ± 2 months old; 264.80 ± 13.75 kg), 40 ½ Angus × ½ Nellore (11 ± 2 months old; 278.00 ± 20.32 kg), and 40 ½ Senepol × ½ Nellore (9 ± 2 months old; 226.70 ± 22.24 kg), were used. The animals were acquired from different herds, and due to this heterogeneity, we chose to use breed as a random effect and thus dilute the variation in response to the high sample number. Before the experiment, all bulls were fed grass without creep-feeding supplementation.

### 2.2. Growing Phase (Growing Feed)

The experiment was conducted in a randomised block design with two supplements, (1) mineral (ad libitum; *n* = 60) and (2) protein + energy (3 g/kg BW/day; *n* = 60), during the growing. The growing phase occurred during the summer season in Brazil (December to May, 155 d). At the beginning of the experiment, the bulls were divided based on BW and placed in one of two treatments during the growing. The supplements were based on tropical conditions [12], and the composition of the diets is presented in Table 1. The mineral premix was added in both treatments (mineral and protein + energy). The amount of supplement was calculated to meet the requirements for an average daily gain of 0.6 kg/d, according to Valadares Filho et al. [13]. During the growing phase, bulls were fed once a day (10:00 h), and the grazing area consisted of Brachiaria grass (*Urochloa brizantha cv.* “Xaraés”) distributed into 12 paddocks (approximately 1.8 ha each), with 10 bulls/paddock. Each paddock had semi-circular drinkers and covered feed troughs (3.0 m × 0.5 m), with easy access to both sides for supplementation. Every 28 days, the bulls were weighed, and their BW was used to adjust the amount of supplement supplied. Mineral feed was available to the bulls ad libitum, and the protein + energy supplement amount was calculated based on BW at the beginning of each experimental period.

### 2.3. Finishing Phase (Second Experimental Phase)

The finishing phase was conducted during the winter and dry seasons (May to September; 129 days). The adaptation period was 20 days, based on the “step-up” procedure, before the second experimental phase. Following the growing (first experimental phase), 30 bulls within each treatment (supplementation) were selected and assigned to one of the two finishing systems: (1) pasture plus concentrate supplementation (20 g/kg BW/day) and (2) feedlot system, where bulls received corn silage as roughage and concentrate (25:75; corn silage:concentrate). The chemical composition and profile of the fatty acids in the experimental diets are shown in Table 1. The amount of supplement was calculated to meet the requirements for an average daily gain of 1.5 kg/d, according to Valadares Filho et al. [13].

All bulls designated for treatment with pasture + supplementation were housed in the same paddock during the finishing phase (12 paddocks, with 5 bulls/paddock from growing feed). Every 28 days, the bulls were weighed, and their BW was used to adjust the amount of concentrate supplemented (20 g/kg BW/day). The bulls of the pasture system were fed concentrate once per day (10:00 h) during the experimental period. The bulls designated for the feedlot system were retained in individual pens of 12 m^2^, partially covered concrete floors, with feed-trough and free water access. The feedlot basal diet comprised 750 g/kg concentrate (corn, soybean meal, and premix) and 250 g/kg roughage (corn silage). The bulls were fed twice per day (08:00 h and 15:00 h), and the amount of diet was adjusted weekly for a 5% feed refusals.

### 2.4. Slaughter Procedure and Muscle Sampling

After 285 days, bulls with BW of 510.90 ± 43.65 kg (Nellore), 532.70 ± 55.81 kg (½ Angus) and 466.20 ± 48.48 kg (½ Senepol) were transported to a commercial slaughterhouse (Minerva Foods, Barretos, São Paulo, Brazil) located 90 km from the experimental area. The bulls were slaughtered based on the usual practices of the Brazilian beef industry according to the Brazilian RIISPOA—Regulation of Industrial and Sanitary Inspection of Animal Products. The carcasses were then divided medially from the sternum to the spine, resulting in two similar halves. After these procedures, muscle samples were collected from the LT muscle of the left half-carcass at the 12th to 13th rib. The muscle samples were frozen in liquid nitrogen and stored at −80 °C at the laboratory for gene expression and enzyme analyses. Subsequently, the half carcasses were washed, identified, and stored in a chilling chamber at 4 °C for 24 h. After chilling, the LT muscle samples were collected from the left side of the carcasses between the 12th and 13th ribs and stored at −20 °C for beef chemical composition and fatty acid analysis. The samples were transported to the Animal Science Laboratory at São Paulo State University (Jaboticabal, Brazil).

### 2.5. Chemical Composition of Beef

To determine the chemical composition of beef, the steaks were thawed at 4 °C for 24 h, ground, and subjected to composition analyses (crude fat, ash, crude protein, and moisture) using the FoodScan Meat Analyser™^®^ (FOSS, Hillerod, Denmark) with a near-infrared spectrophotometer (analyses AOAC method: 2007-04).

### 2.6. Fatty Acid Profile of Beef and Diet

Sample lipids were extracted according to Bligh and Dyer [14]. Briefly, 15 g of meat sample was subjected to extraction with a chloroform–methanol mixture (2:1 ratio) and then transmethylated [15]. A 1 μL aliquot of transmethylated lipid was injected into a gas chromatograph (Shimadzu GC-2010 Plus; Shimadzu Corporation, Kyoto, Japan) with a flame ionisation detector and capillary column (Restek-RT^®^ 2560, Bellefonte, PA, USA; 100 m long, 0.25 mm internal diameter, and 0.20 μm film thickness). Hydrogen was used as the carrier gas at a flow rate of 1.0 mL/min. The temperature program of the oven of the gas chromatograph began at 100 °C with a standby time of 5 min and then increased to 240 °C (4 °C/min) with a standby time of 20 min. The detector temperature was 260 °C. Identification and quantification of the proportion of fatty acids were performed by comparing their retention times with those of commercial standards of total fatty acid methyl esters (Supelco 37 component FAME mix; conjugated linoleic acid methyl ester (trans10–cis12) and conjugated linoleic acid methyl ester (cis9–trans11); Sigma-Aldrich, Bellefonte, PA, USA). The results are expressed as mg/100 g of beef.

### 2.7. Lipogenic Enzyme Activity

Approximately 1.5 g of LT was cut and placed in 4.5 mL of 0.1 M phosphate buffer (K_2_HPO_4_, pH 7.4, 25 °C), homogenised, and centrifuged at 3000× *g* for 15 min at 4 °C. The pellet was then discarded, and the supernatant was centrifuged at 15,000× *g* for 30 min at 4 °C. The resulting supernatant fractions were used for enzyme measurements. NADP-malate dehydrogenase and isocitrate dehydrogenase enzyme activity were measured as described by Martin [16] and Bergmeyer and Bernt [17,18], respectively. All enzyme assays were performed in duplicate using the spectrophotometric absorbance of the solutions in cuvettes at 340 nm. The slopes of the linear rates of NADPH production were used to calculate enzyme activity.

### 2.8. Gene Expression Analyses

The target and reference primers were designed using sequences registered and published in the GenBank public data bank of the National Center for Biotechnology Information platform (Table 2). Primers were designed using OligoPerfect Designer software (Invitrogen, Karlsruhe, Germany) and synthesized (Invitrogen, Carlsbad, CA, USA). Nine target genes (*PPARG*, *SREBP-1c*, *SCD1*, *ACCA*, *LPL*, *FABP4*, *ACOX*, *CPT2*, and *PPARA*) and two target reference genes (*β-actin* and glyceraldehyde-3-phosphate dehydrogenase (*GAPDH*)) were used, as proposed by Vandesompele et al. [19]. Total RNA extraction was performed using a RNeasy Lipid Tissue Mini Kit (Qiagen, Valencia, CA, USA). RNA contamination (260/280 and 260/230) and concentration (ng/μL) were assessed using a NanoDrop 1000 Spectrophotometer (Thermo Fisher Scientific, Santa Clara, CA, USA, 2007). RNA quality was assessed using an Agilent 2100 Bioanalyzer (Agilent, Santa Clara, CA, EUA, 2009) and the Agilent RNA 6000 Nano Chip kit (Agilent, Santa Clara, CA, USA).

The cDNA synthesis was performed using SuperScript III First-Strand Synthesis SuperMix for qRT-PCR (Invitrogen, Carlsbad, CA, USA), according to the manufacturer’s instructions. A 7500 Real-Time PCR system (Applied Biosystems, Foster, CA, USA, 2009) was used for qPCR with a SYBR Green RT-PCR kit from Bio-Rad. The cycling conditions were 2 min polymerase activation at 95 °C and 40 cycles at 95 °C for 15 s and 60 °C for 30 s. A validation assay of amplification efficiencies demonstrated that the target and reference genes were approximately equivalent. Relative mRNA expression was calculated according to ΔC_T_ = C_T_ (target gene) − C_T_ (average reference genes). The calibration was determined using the formula ΔΔC_T_ = ΔC_T_ (sample) − ΔC_T_ (calibrator), and the mineral finished in pasture plus supplementation treatment was used for each breed. Relative expression was evaluated using the 2^−ΔΔCT^ formula [20].

### 2.9. Gene Set Enrichment Analysis

The ClueGO of the Cytoscape program 3.7.1 was used for the enrichment analysis with the genes studied using the bovine genome UMD 3.1. An enrichment analysis was performed to visualise non-redundant biological terms for genes using the ClueGO plug-in of Cytoscape [21], with bovine genome UMD 3.1 (http://www.ncbi.nlm.nih.gov/genome/?term=bos+taurus, accessed on 22 August 2019) as reference. The genes were enriched considering the gene ontology (GO) biological processes classification system.

### 2.10. Statistical Analysis

The experimental design was a completely randomised block (by weight: light and heavy; and by breed: Nellore, ½ Angus, and ½ Senepol) in a 2 × 2 factorial arrangement, with two supplements administered during the growing (mineral or protein + energy supplementation) and two finishing systems (pasture and feedlot systems). All data were analysed using PROC MIXED software of SAS 9.4 (SAS Inst. Inc., Cary, NC, USA, Cary Inc., Rural Hall, NC, USA). The statistical model included treatments and all interactions as fixed effects and bulls nested within the paddock and breed as random effects. The mean and standard error of the mean were calculated for each variable (the experimental unit was the animal; *n* = 30/ treatment). When significant main or interaction effects were detected, Tukey’s test (*p* ≤ 0.05) was used to determine the differences between means.

## 3. Results

### 3.1. Meat Composition

The feed strategy in the growing (mineral vs. protein + energy supplement) did not result in higher lipid content in beef (*p* ≥ 0.05). At the end of the finishing phase, the cattle fed with mineral or protein + energy had lipid averages of 2.03 and 2.19 g/100 g of beef, respectively (Table 3). The finishing system affected the lipid content (*p* < 0.001) and moisture (*p* < 0.001; Table 3). The meat of bulls finished in the feedlot system had an 86.48% higher lipid concentration than meat from bulls finished in the pasture system (2.75 vs. 1.48 g/100 g of beef, respectively). The increase in lipid content resulted in a decrease (*p* ≤ 0.001) in the moisture content of beef from the feedlot group compared to beef from the pasture group, with averages of 72.55 vs. 73.74 g/100 g, respectively. The animals presented beef with similar ash and protein contents (*p* > 0.050; Table 3). No interaction between the growing feed and finishing system was observed (*p* > 0.050) for the chemical composition.

### 3.2. Fatty Acid Profile and Enzyme

In the current study, the total saturated fatty acid (SFA) profile was not affected (*p* > 0.050) during the growing feed (Table 4). A higher total concentration of SFA (*p* = 0.003) was observed in the meat of bulls finished in the feedlot system, which was due to the increase (*p* ≤ 0.003) in myristic (C14:0), palmitic (C16:0), and margaric (C17:0) fatty acids found in this group (Table 4).

The total MUFA (Table 4) increased in the meat of bulls from the feedlot system compared to the pasture system (*p* = 0.001; 903.95 vs. 645.01 mg/100 g beef, respectively). In addition, increases (*p* ≤ 0.010) in the palmitoleic (C16:1), heptadecenoic (C17:1), and oleic (C18:1n9c) acid content were observed in the meat of the bulls from the feedlot compared to the pasture finishing system (59.27 vs. 43.26, 9.72 vs. 7.49, and 827.65 vs. 583.65, mg/100 g beef, respectively; Table 4). In the current study, the pasture group presented higher (*p* ≤ 0.003) arachidonic (C20:4n6) and dihomo-γ-linolenic (C20:3n6) fatty acid levels than the feedlot system (26.76 vs. 20.17; 5.38 vs. 4.21 mg/100 g beef, respectively; Table 4). The meat of the bulls from the pasture system had (*p* ≤ 0.006) higher linoleic (C18:2n6) and α-linolenic (C18:3n3) fatty acid concentration (128.04 vs. 107.57; 7.56 vs. 6.32 mg/100 g beef, respectively; Table 4).

A similar concentration in C18:2 trans10–cis12 fatty acid was observed between treatments (*p* > 0.050). The pasture system with intensive supplementation increased in n6 total and n6:n3 ratio (*p* ≤ 0.009; 162.66 vs. 134.76; 14.63 vs. 12.72 mg/100 g meat, respectively) and tended (*p* = 0.090; 10.97 vs. 9.98 mg/100 g meat, respectively) to provide greater n3 total concentrations in beef compared to the feedlot system. An interaction (*p* = 0.021) was observed between the growing feed and finishing system for C18:2 cis9–trans11.

The growing feed or finishing system did not alter (*p* ≥ 0.050) the NADP–Malate dehydrogenase enzyme activity. Isocitrate dehydrogenase enzyme activity was lower (*p* = 0.020) in the meat of bulls from feedlot than in the pasture finishing system (2788.07 vs. 3279.54 nmol/min, respectively; Table 5).

### 3.3. Gene Expression

**Figure 1:**
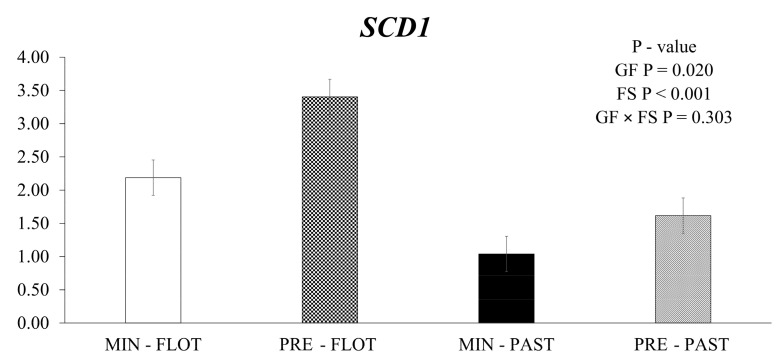
Relative expression of *SCD1* (*stearoyl-CoA desaturase*) in the *Longissimus thoracis* muscle from young bulls fed mineral (ad libitum; MIN) or protein + energy (3 g/kg BW/day; PRE) during the growing and finished in pasture (20 g/kg BW/day of concentrate; PAST) or feedlot (75:25 corn silage:concentrate ratio; FLOT). GP = Growing feed; FS = Finishing system.

The muscle of bulls fed the feedlot system had greater (*p* < 0.001) expression levels of *stearoyl-CoA desaturase* (*SCD1*) compared to the muscle of bulls from the pasture system (Figure 1). In addition, the animals fed protein + energy during the growing presented higher (*p* = 0.020) *SCD1* expression compared to animals that received mineral supplementation.

**Figure 2:**
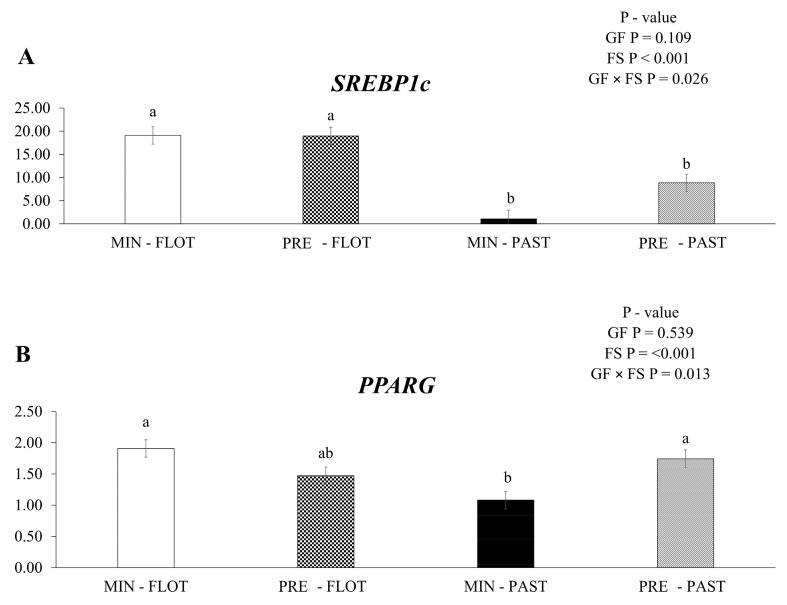
Relative expression of *SREBP-1c* (*sterol regulatory element-binding protein-1c*); (**A**) and *PPARG* (*peroxisome proliferator-activated receptor gamma*); (**B**) in the *Longissimus thoracis* muscle from young bulls fed mineral (ad libitum; MIN) or protein + energy (3 g/kg BW/day; PRE) during the growing and finished in pasture (20 g/kg BW/day of concentrate; PAST) or feedlot (75:25 corn silage:concentrate ratio; FLOT). GP = Growing feed; FS = Finishing system. The means without a common letter are different (*p* < 0.050).

An interaction among growing feed and finishing system (*p* = 0.026) was observed, with *sterol regulatory element-binding protein-1c* (*SREBP1c*) having lower mRNA expression in the muscle of bulls finished in the pasture system regardless of the supplementation during the growing phase (Figure 2A). In addition, lower (*p* = 0.013) *peroxisome proliferator-activated receptor gamma* (*PPARG*) mRNA expression was detected in the muscle of bulls fed mineral during the growing phase and finished in the pasture system (Figure 2B).

**Figure 3:**
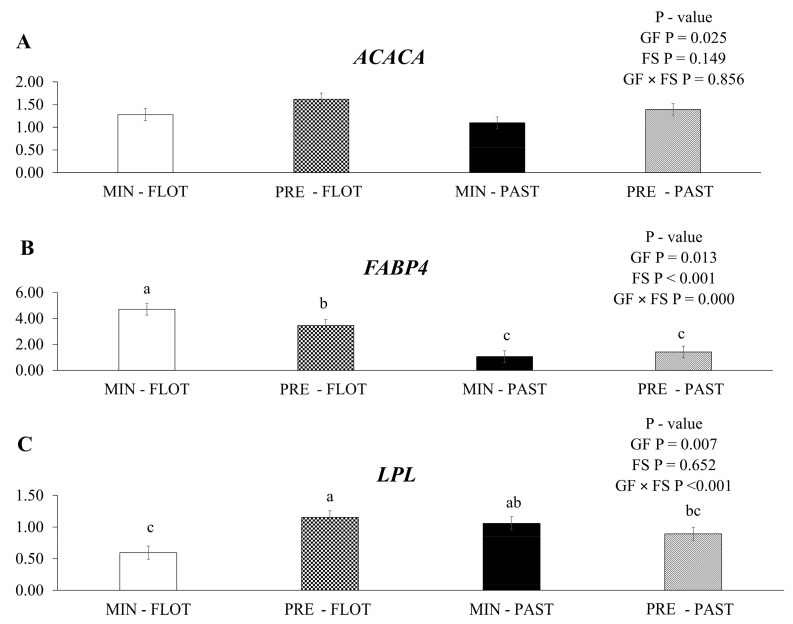
Relative expression of *ACACA* (*acetyl CoA carboxylase alfa*); (**A**), *FABP4* (*fatty acid binding protein 4*); (**B**) and *LPL* (*lipoprotein lipase*); (**C**) in the *Longissimus thoracis* muscle from young bulls fed mineral (ad libitum; MIN) or protein + energy (3 g/kg BW/day; PRE) during the growing and finished in pasture (20 g/kg BW/day of concentrate; PAST) or feedlot (75:25 corn silage:concentrate ratio; FLOT). GP = Growing feed; FS = finishing system. The means without a common letter are different (*p* < 0.050).

The expression of *acetyl CoA carboxylase alfa* (*ACACA*) mRNA was upregulated (*p* = 0.025; Figure 3A) in the muscle of bulls fed protein + energy compared to mineral feed, regardless of the finishing system (1.51 vs. 1.19). An increase in *ACACA* mRNA expression in the muscle of bulls from the feedlot finishing system compared to the pasture finishing system was not observed (*p* = 0.149).

In the current study, an interaction (*p* ≤ 0.001) was found between the growing feed and finishing system for *fatty acid binding protein 4* (*FABP4*) mRNA (Figure 3B). Greater expression was detected in the muscle of bulls fed mineral during the growing and finished in the feedlot system. Moreover, bulls fed with protein + energy supplement followed by the feedlot system had intermediate values, and the two lowest values were found in the muscle of bulls finished in the pasture system regardless of the feed during the growing (mineral or protein + energy) (4.71, 3.47, 1.06, and 1.41, respectively).

An interaction (*p* ≤ 0.001) between the growing feed and finishing systems was observed for the *lipoprotein lipase* (*LPL*) mRNA expression (Figure 3C). The muscle of bulls from protein + energy during the growing and finished in the feedlot system had higher levels of *LPL* mRNA expression than the muscle of bulls fed mineral during the growing and finished in the feedlot system.

**Figure 4:**
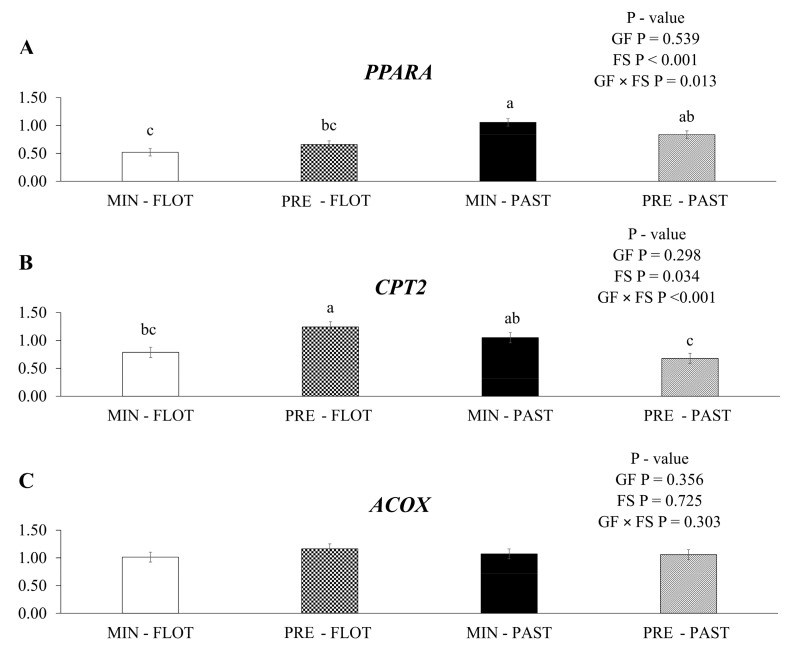
Relative expression of *PPARA* (*peroxisome proliferator-activated receptor alfa*); (**A**), *CPT2* (*carnitine palmitoyl transferase 2*); (**B**) and *ACOX* (*acyl CoA oxidase 1*); (**C**), in the *Longissimus thoracis* muscle from young bulls fed mineral (ad libitum; MIN) or protein + energy (3 g/kg BW/day; PRE) during the growing phase and finished in pasture (20 g/kg BW/day of concentrate; PAST) or feedlot (75:25 corn silage:concentrate ratio; FLOT). GP = Growing feed; FS = Finishing system. The means without a common letter are different (*p* < 0.050).

In this study, an interaction (*p* = 0.013) was found in the muscle of bulls between the growing and finishing systems (Figure 4A) for *peroxisome proliferator-activated receptor alfa* (*PPARA*) mRNA expression, where this gene was upregulated in the muscle of bulls fed with mineral during the growing and finished in the pasture system.

An interaction between the growing feed and finishing systems was observed for the gene encoding *carnitine palmitoyl transferase 2* (*CPT2*; *p* < 0.001; Figure 4B). The muscle of bulls fed protein + energy during the growing and finished in the feedlot system had higher mRNA expression of the *CTP2* gene than the muscle of bulls fed protein + energy and finished in the pasture system. In contrast, the muscle of bulls fed mineral and finished in the feedlot or pasture system had intermediate *CPT2* mRNA expression (1.24, 0.68, 0.79, and 1.05, respectively). No treatment effects (*p* ≥ 0.050) or interactions were found for *acyl CoA oxidase 1* (*ACOX,* Figure 4C).

## 4. Discussion

To the best of our knowledge, this is the first study to investigate the relative expression of genes related to lipid metabolism in the intramuscular adipose tissue of LT muscle from cattle supplemented during the growing phase and finished in the tropical pasture or feedlot with intensive supplementation. We hypothesized that the dietary treatment with protein + energy during the growing followed by the feedlot finishing system could increase lipogenic genes and decrease lipolytic genes, resulting in increased lipid content in the meat of bulls. However, our hypothesis was not confirmed, which can be explained by the hot carcass weight (HCW) and the degree of marbling score [22] that the animals reached. At the end of the growing phase, the animals from the protein + energy group presented 21.9 kg body weight more than the mineral group (data not presented). However, this difference was diluted during the finishing phase (120 days), where the bulls had a similar value of HCW (284 and 291 kg for protein + energy and Mineral, respectively).

The bulls from the pasture system have more significant energy expenditure when compared to the feedlot system, which reduces energy available required for lipid deposition in meat. Animals in the pasture system have a higher energy expenditure in response to more activity related to feeding even when they are administered the concentrate [23]. Overall, the bulls require greater movement (physical activity), collection, and selection of pasture (source of roughage), which promotes greater expenditure and energy requirement than those in the feedlot [8]. In addition, animals on pasture showed an increase of 11% in metabolic energy requirements for maintenance when compared to the animals in the feedlot system [24].

The fatty acid profile was examined in the sample collected at the end of the finishing phase, which had a 129-day duration, long enough to change the fatty acid profile. Lipogenesis occurs through dietary lipid absorption and de novo fatty acid synthesis in animals [5]. The increase in these fatty acids in beef from the feedlot group, where the corn silage diet had lower total SFA than the pasture composition (Table 1), could be due to an increase in de novo fatty acid synthesis and higher enzyme activity of fatty acid synthase, a key enzyme in the lipogenic pathway that catalyses the reactions of fatty acid biosynthesis and conversion of acetyl-CoA and malonyl-CoA to palmitic acid, which may have increased the isomers of SFA [25].

Isocitrate dehydrogenase plays a crucial role in lipid metabolism, catalysing the conversion of oxidative decarboxylation of isocitrate to α-ketoglutarate with the production of NADPH [26] for de novo fatty acid synthesis. The higher values found in isocitrate dehydrogenase enzyme activity suggest that the feedlot system can provide higher energy, as this enzyme can be reduced by increasing the energy levels [27].

**Figure 5:**
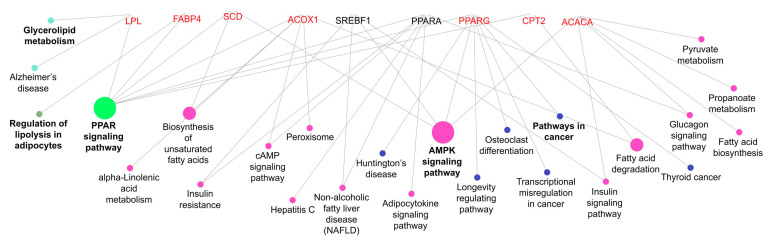
Functional classification of the lipid metabolism genes for the biological process GO category. The edges represent the interaction between the genes and processes. The node fill color represents the relationship of genes caused by a common process (i.e., purple nodes represent the process associated with two or more genes and green node represents the process with the most relationship). The genes are grouped by a common process.

The high concentrations of oleic and palmitoleic acid in the meat of bulls from the feedlot system are related to *SCD1* expression (∆9 desaturase enzyme). *SCD1* is associated with the biosynthesis of unsaturated fatty acids (Figure 5) and is a key enzyme that catalyses the desaturation of a range of fatty acyl-CoA substrates, mainly palmitoyl and stearoyl, resulting in palmitoleic and oleic acid, respectively [28]. According to Smith et al. [29], the accumulation of MUFA in adipose tissues coincides with an increase in *SCD1* gene expression. The increase in monounsaturated acid, such as oleic acid, is related to meat palatability [30], and oleic fatty acid represents the largest amount of monounsaturated acid in beef [31], ultimately aligning with our results, regardless of the treatments.

The meat of cattle fed exclusively with grass or grass plus supplements had greater α-linolenic fatty acid content [32], while the beef of animals fed grain had higher linoleic content due to diet composition. In the current study, the pasture group presented higher linolenic fatty acid levels and, interestingly, higher linoleic concentration than the feedlot system, which was not expected, suggesting that the bulls finished in the feedlot system could have higher ruminal biohydrogenation activity, leading to a decrease in linoleic acid in beef. Bulls finished in the pasture system had a higher decline and variation in ruminal pH once the concentrate supplementation was offered separately from roughage, which may have resulted in a reduction in microorganisms that play an important role in biohydrogenation [33].

γ-Linolenic acid, eicosatrienoic acid (C20:2n6), and arachidonic acid are produced from linoleic acid by the action of desaturase and elongase enzymes, whereas eicosapentaenoic acid (C20:5n3) and docosahexaenoic acid (C22:6n3) are produced from alpha-linolenic acid [34]. In this context, the increase in linoleic acid in the meat of bulls from the pasture system aligned with the increase in arachidonic and dihomo-γ-linolenic fatty acids, which was not expected but could have caused an increase in n6:n3 ratio values due to a higher n6 total concentration in this group. The lower n6:n3 ratio is recommended for the benefits of human health; it is found to be around 1 for beef produced using the grass diet [35,36,37]. The n6:n3 ratio in beef increases with the increased inclusion of concentrate supplements in grazing animal diets [38]. Although some studies show that concentrate supply has not changed n6:n3 in the meat, these works have tested concentrate inclusion in up to 50% of the diet. In this sense, in the present study, this change may be related to the high supply of concentrate (2% BW) for animals in the pasture system.

The C18:2 trans10–cis12 can be produced because of rumen pH reduction as a grain-based diet and can decrease the relative abundance of *SREBP1c*, a gene responsible for encoding sterol regulatory element-binding protein [38,39], consequently contributing to a reduction in fat deposition. However, this result was not found in the current study, where C18:2 trans10–cis12 in the meat of bulls had a similar concentration, which means that it was not related to changes in *SREBP1c* activity.

In this way, some n3 fatty acid isomers, such as arachidonic and docosahexaenoic acid, can affect the expression of genes related to lipid metabolism by controlling *SREBP1c*, which is the main gene controlling lipogenesis [40]. Therefore, the pasture system with intensive supplementation tended to provide greater n3 concentrations in beef, which may have helped to decrease intramuscular fat concentrations.

The expression levels of *SREBP1c* are related to energy availability, and it is a major factor in the expression of genes related to fat deposition [41]. Although not measured in this study, the mineral feed during the growing phase followed by the pasture finishing system may have provided lower blood insulin and glucose concentrations. Accordingly, the decrease in insulin may have decreased the concentration of *SREBP1c*, as this gene is associated with two pathways (Figure 5), insulin signalling and AMPK signalling. Such findings indicate that insulin can control *SREBP1c* abundance and induce de novo lipogenesis [42] and agree with the results of a higher concentration of isocitrate dehydrogenase enzyme activity in the mineral feed during the growing followed by the pasture finishing system (3520.39 nmol/min). Moreover, such results also help explain the *SCD1* gene expression in our study, considering that *SCD1* activity is increased by dietary glucose, fructose, and insulin [43].

In addition, the above explanation can be related to lower levels of *PPARG* expression in the muscle of bulls fed mineral during the growing phase and finished in the pasture system. *PPARG* can regulate biological processes, such as lipid metabolism; however, it is more highly expressed in adipose tissue and participates in adipogenesis and insulin sensitivity [44]. The *PPARG* is linked to *SREBP1c* and *ACACA* by the AMPK signalling pathways (Figure 5).

*ACACA* is associated with the biosynthesis of fatty acids and it is involved in the first step of SFA synthesis and the enzyme carboxylation of acetyl-CoA into malonyl-CoA in response to diet and hormones [45]. An increase in *ACACA* mRNA expression in the muscle of bulls from the feedlot group was expected but was not observed. Thus, the possible increase in SFA by de novo fatty acid synthesis in the feedlot group may occur due to the increased activity of other enzymes, such as fatty acid synthase; however, this enzyme was not measured in this study. The expression levels of *ACACA* and fatty acid synthase are related to fatty acid biosynthesis and are not regulated in coordination [38].

The transport of fatty acids into cells is facilitated by *FABP4* [46]; thus, the increase in its gene expression can be related to an increase in triacylglycerols from diets. The lowest *FABP4* expression in the muscle of bulls finished in the pasture group, regardless of the growing supplementation, may have been due to the lower intramuscular fat of this group. Yang et al. [47] found an increase in *FABP4* levels when intramuscular fat was increased by a higher-energy diet compared to a lower-energy diet.

Although *LPL* and *FABP4* can exhibit complementary functions [38], there is no similar relationship between the expression of both genes due to sampling of both adipocytes, which may express more FABP4, and myocytes, which may express more *LPL*. Lipoprotein lipase is an enzyme that catalyses the hydrolysis of triglycerides present in lipoproteins [47]; thus, the muscle of bulls fed protein + energy and finished in the feedlot system may have higher lipid turnover and higher triglyceride hydrolysis into non-esterified fatty acids to supply energy for tissues, which leads to increased gene expression.

Although *PPARA* is highly expressed in the liver [48], this enzyme is responsible for peroxisome proliferator-induced responses, including the transcriptional activation of genes involved in fatty acid oxidation [44]. In this study, the less *PPARA* expression in the muscle of bulls fed with mineral supplementation during the growing followed by the pasture finishing system, suggesting that the muscle of these bulls had a higher lipid oxidation rate. The *CPT2* and *ACOX1* are other genes that may participate in fatty acid oxidation mechanisms in muscle and are related to the fatty acid degradation pathway (Figure 5); however, no change was observed in *ACOX1* mRNA expression. In this study, higher lipid content was expected in meat from animals fed protein + energy during the growing and finished in the feedlot; however, the up-regulated expression of *CPT2* in these animals may have influenced the degree of marbling, considering the role of this enzyme in mitochondrial long-chain fatty acid oxidation [49].

Fat deposition occurs in response to the activation of metabolic actions, such as lipogenesis and lipolysis, which occur according to the need for lipid release or storage, as well as the interaction of dietary energy and the level of expression of the gene’s relationship with lipids [41]. Accordingly, even if the hypothesis of the study was not confirmed, the supplementation strategy during the growing phase affected gene expression but did not result in higher lipid content at slaughter.

## 5. Conclusions

During the growing phase, the supplementation strategy of bulls showed changes in the lipolytic and lipogenic genes, but not enough to cause changes in intramuscular fat at the finishing stage. The finishing system impacted the genes and affected the intramuscular fat and fatty acid. In addition, our results indicate that beef from pasture-fed bulls supplemented with concentrate had a greater concentration of total saturated and a higher n6:n3 ratio in meat, which is considered non-ideal for human health.

## Figures and Tables

**Table 1:** Chemical composition of the experimental diets.

Items	Growing		Finishing		
	PRE ^4^	Pasture	Concentrate	Pasture	Corn Silage
Ingredients, g/kg DM					
Corn	735	-	7890	-	-
Soybean meal	106	-	1650	-	-
Mineral premix ^1^	159	-	46.00	-	-
*Chemical composition*					
Dry matter	860	332	899	458	301
Organic matter	892	925	910	923	950
Crude protein	205	128	160	113	95.0
Neutral detergent fibre	265	575	251	582	331
Ether extract	63.0	24.1	66.0	23.0	71.0
Fatty acid, g/100 g of total FA ^3^					
Myristic (C14:0)	0.08	1.30	0.08	3.09	0.27
Palmitic (C16:0)	11.2	36.5	11.2	35.3	17.6
Margaric (C17:0)	0.09	0.49	0.09	0.65	0.22
Stearic (C18:0)	3.94	3.60	3.76	4.16	3.46
C20:0	0.38	0.99	0.38	1.71	0.84
C22:0	0.45	1.28	0.44	2.03	0.44
C24:0	0.18	2.12	0.20	3.02	0.78
Palmitoleic (C16:1)	0.12	0.46	0.09	0.42	0.23
Oleic (C18:1n9c)	28.4	4.35	29.9	6.17	34.2
Linoleic (C18:2n6c)	48.6	14.8	47.6	14.1	36.5
α-Linolenic (C18:3n3)	4.62	30.3	4.40	22.6	3.69
SFA ^2^	16.3	46.3	16.1	49.9	23.7
MUFA ^2^	28.5	4.81	29.9	6.59	34.4
PUFA ^2^	53.2	45.1	52.1	36.6	40.2

^1^ Sodium 80 g/kg; Calcium 153 g/kg; Phosphorus 30 g/kg; Sulfur 30 g/kg; Zinc 1925 mg/kg; Copper 520 mg/kg; Manganese 400 mg/kg; Iodine 30 mg/kg; Cobalt 38 mg/kg; Selenium 10 mg/kg; Vit A 55,000 Ul/kg; Vit D3 7500 Ul/kg; Vit E 750 Ul/kg; Monensin 400 mg/kg; NNP 620 g /kg; ^2^ SFA = saturated fatty acid; MUFA = monounsaturated fatty acids; PUFA = polyunsaturated fatty acids. ^3^ Fatty acid; ^4^ Protein + energy (3 g/kg BW/day).

**Table 2:** Primer sequences used for quantitative RT-PCR analyses.

Gene Abbrevation	Gene	Primer	R^2^	Efficiency
*PPARG*	*Peroxisome proliferator-activated receptor gamma*	F: CGATATCGACCAACTGAACC	0.992	90.788
		R: AACGGTGATTTGTCTGTCGT		
*SREBP-1c*	*Sterol regulatory element-binding protein-1c*	F: GAGCCACCCTTCAACGAA	0.999	100.593
		R: TGTCTTCTATGTCGGTCAGCA		
*SCD1*	*Stearoyl-CoA desaturase*	F: TTATTCCGTTATGCCCTTGG	0.997	94.776
		R: TTGTCATAAGGGCGGTATCC		
*ACACA*	*Acetyl CoA carboxylase alfa*	F: TGAAGAAGCAATGGATGAACC	0.998	101.32
		R: TTCAGACACGGAGCCAATAA		
*LPL*	*Lipoprotein lipase*	F: CTCAGGACTCCCGAAGACAC	0.993	94.257
		R: GTTTTGCTGCTGTGGTTGAA		
*FABP4*	*Fatty acid binding protein 4*	F: GGATGATAAGATGGTGCTGGA	0.997	90.259
		R: ATCCCTTGGCTTATGCTCTCT		
*ACOX*	*Acyl-CoA oxidase 1*	F: GCTGTCCTAAGGCGTTTGTG	0.991	90.993
		R: ATGATGCTCCCCTGAAGAAA		
*CPT2*	*Carnitine Palmitoyltransferase 2*	F: CATGACTGTCTCTGCCATCC	0.991	94.577
		R: ATCACTTTTGGCAGGGTTCA		
*PPARA*	*Peroxisome proliferator-activated receptor alfa*	F: CAATGGAGATGGTGGACACA	0.994	91.665
		R: TTGTAGGAAGTCTGCCGAGAG		
*β-Actin*	*β-actin*	F: GTCCACCTTCCAGCAGATGT	0.998	93.059
		R: CAGTCCGCCTAGAAGCATTT		
*GAPDH*	*Glyceraldehyde 3 phosphate*	F: CGACTTCAACAGCGACACTC	0.994	92.896
		R: TTGTCGTACCAGGAAATGAGC		

**Table 3:** Hot carcass weight and chemical composition (lipid, ash, protein and moisture [g/100 g of meat]) of *Longissimus thoracis* meat from young bulls supplemented with mineral or protein + energy during the growing phase and finished in pasture plus concentrate or feedlot system.

Finishing System	Feedlot ^1^		Pasture ^2^		SEM ^5^	GF ^6^	FS ^7^	GF × FS
Growing Feed	MIN ^3^	PRE ^4^	MIN ^3^	PRE ^4^				
Hot carcass weight, kg	297	297	272	285	13.10	0.124	<0.001	0.096
Lipid	2.66	2.84	1.41	1.54	0.180	0.466	<0.001	0.868
Ash	2.22	2.48	2.64	2.53	0.128	0.623	0.071	0.150
Protein	22.3	22.4	22.0	22.4	0.199	0.255	0.370	0.424
Moisture	72.8	72.3	73.9	73.6	0.296	0.129	<0.001	0.826

^1^ Feedlot (75:25 corn silage:concentrate ratio); ^2^ Pasture (20 g/kg BW/day of concentrate); ^3^ Mineral (ad libitum); ^4^ Protein + energy (3 g/kg BW/day); ^5^ Standard error of mean; ^6^ Growing feed; ^7^ Finishing system.

**Table 4:** Effect of mineral or protein + energy supplement during the growing phase and finishing with intensive supplementation in pasture plus concentrate or feedlot system on fatty acid profile (mg/100 g of meat) from *Longissimus thoracis* of young bulls.

Finishing System	Feedlot ^1^		Pasture ^2^		SEM ^5^	GF ^6^	FS ^7^	GF × FS
Growing Feed	MIN ^3^	PRE ^4^	MIN ^3^	PRE ^4^				
C12:0	0.92	0.64	0.62	0.67	0.108	0.339	0.241	0.064
C14:0	62.90	45.04	32.14	34.80	6.824	0.300	0.001	0.060
C15:0	4.94	4.21	4.04	4.26	0.563	0.854	0.440	0.211
C16:0	614.60	495.97	352.85	354.25	61.644	0.453	0.000	0.249
C17:0	19.69	15.69	12.33	12.46	2.202	0.432	0.003	0.222
C18:0	424.43	364.77	306.54	324.67	49.170	0.948	0.068	0.338
C14:1	14.27	9.30	8.87	8.51	1.840	0.136	0.056	0.110
C15:1	2.20	1.84	1.88	1.99	0.265	0.995	0.959	0.275
C16:1	65.54	53.00	44.79	41.72	7.339	0.347	0.010	0.373
C17:1	10.38	9.07	7.81	7.17	1.026	0.307	0.005	0.533
C18:1n9c	857.23	798.07	584.83	582.47	85.856	0.873	0.000	0.491
C18:2c9–t11	62.24	32.73	25.67	47.44	15.630	0.678	0.366	0.021
C18:2t10–c12	14.93	13.26	15.55	14.37	1.247	0.231	0.267	0.694
C18:2n6c	107.57	107.58	131.22	124.87	10.954	0.838	0.006	0.555
C18:3n6	1.05	0.95	1.09	1.00	0.079	0.192	0.334	0.972
C18:3n3	6.11	6.53	7.90	7.22	0.606	0.675	0.001	0.261
C20:2n6	1.67	1.70	1.46	1.38	0.124	0.957	0.005	0.727
C20:3n6	4.11	4.30	5.75	5.01	0.545	0.504	0.003	0.182
C20:3n3	0.32	0.25	0.24	0.27	0.040	0.486	0.481	0.102
C20:4n6	19.00	21.33	27.77	25.75	2.560	0.922	0.000	0.122
C20:5n3	2.45	2.52	2.70	2.74	0.229	0.777	0.164	0.778
C22:6n3	1.35	0.60	0.42	0.62	0.346	0.509	0.133	0.119
∑SFA ^8^	1129.55	926.32	708.52	740.42	117.232	0.648	0.003	0.228
∑MUFA ^9^	936.63	871.28	648.18	641.85	93.766	0.849	0.001	0.502
∑PUFA ^10^	239.88	194.06	220.18	230.67	18.862	0.245	0.382	0.060
∑n6–PUFA ^11^	133.65	135.86	167.30	158.02	13.799	0.866	0.003	0.425
∑n3–PUFA ^12^	9.86	10.09	11.08	10.85	0.783	0.993	0.090	0.673
n6:n3	12.02	13.42	14.55	14.70	0.929	0.286	0.009	0.312
PUFA:SFA	0.24	0.23	0.33	0.32	0.034	0.403	0.001	0.997

^1^ Feedlot (75:25 corn silage:concentrate ratio); ^2^ Pasture (20 g/kg BW/day of concentrate); ^3^ Mineral (ad libitum); ^4^ Protein + energy (3 g/kg BW/day); ^5^ Standard error of mean; ^6^ Growing feed; ^7^ Finishing phase; ^8^ Sum of 12:0, 14:0, 15:0, 16:0, 17:0 and 18:0; ^9^ Sum of 14:1, 15:1, 16:1, 17:1 and 18:1; ^10^ Sum of 18:2 c9–t11, 18:2 t10–c12, 18:2n6, 18:3n6, 18:3n3, 20:2n6, 20:3n6, 20:3n3, 20:4n6, 20:5n3 and 22:6n3; ^11^ Sum of 18:2n6, 18:3n6, 20:2n6, 20:3n6 and 20:4n6; ^12^ Sum of 18:3n3, 20:3n3, 20:5n3 and 22:6n3.

**Table 5:** NADP–malate dehydrogenase and isocitrate dehydrogenase (nmol/min) in the *Longissimus thoracis* muscle from young bulls fed mineral or protein + energy supplement during the growing phase and finished in pasture or feedlot.

Finishing System	Feedlot ^3^		Pasture ^4^		SEM ^7^	GF ^8^	FS ^9^	GF × FS
Growing Feed	MIN ^5^	PRE ^6^	MIN ^5^	PRE ^6^				
Isocitrate ^1^	2645.6	2930.5	3520.4	3038.7	245.0	0.820	0.020	0.066
NADP-Malate ^2^	47.2	46.5	48.6	43.6	3.8	0.472	0.888	0.554

^1^ Isocitrate dehydrogenase; ^2^ NADP–Malate dehydrogenase; ^3^ Feedlot (75:25 corn silage:concentrate ratio); ^4^ Pasture (20 g/kg BW/day of concentrate); ^5^ Mineral (ad libitum); ^6^ Protein + energy (3 g/kg BW/day); ^7^ Standard error of mean; ^8^ Growing feed; ^9^ Finishing system.

## Data Availability

All relevant data are presented within the paper.
